# *Thecacera sesama* sp. nov. (Nudibranchia, Polyceridae) from Taiwan, evident from morphology and phylogenetic analyses of the 16S rDNA and cytochrome c oxidase I gene

**DOI:** 10.3897/zookeys.1279.184298

**Published:** 2026-05-11

**Authors:** Ho-Yeung Chan, Chen-Lu Lee, Wei-Cheng Chen, Chia-Hao Chang, Yi-Ta Shao, Ka-Lai Pang

**Affiliations:** 1 Institute of Marine Biology and Center of Excellence for the Oceans, National Taiwan Ocean University, Keelung, Taiwan Biology Department, National Museum of Natural Science Taichung Taiwan; 2 Biology Department, National Museum of Natural Science, Taichung, Taiwan Department of Science Education, National Taipei University of Education Taipei Taiwan; 3 Department of Science Education, National Taipei University of Education, Taipei, Taiwan Institute of Marine Biology and Center of Excellence for the Oceans, National Taiwan Ocean University Keelung Taiwan

**Keywords:** bryozoans, COI, cryptic diversity, Heterobranchia, phylogeny, systematics, taxonomy

## Abstract

*Thecacera sesama* Chan & Lee, **sp. nov**. (Nudibranchia, Polyceridae) is described from north-eastern Taiwan based on an integrative taxonomic approach combining morphological and molecular data. The new species is distinguished from its congeners by a unique colour pattern consisting of a translucent white body covered with numerous small, round, black pigment spots and fewer, larger, yellow spots and five gills. While sharing a similar spotted colour pattern with *Thecacera pennigera*, the new species can be clearly distinguished by its significantly smaller body size (maximum length < 3 mm). Phylogenetic analyses of two mitochondrial genes, cytochrome c oxidase subunit I (COI) and 16S rRNA, confirmed it as a new species in *Thecacera*. The molecular data places *Thecacera sesama***sp. nov**. as a sister species to *Thecacera picta*, with a significant interspecific COI divergence of 14.17%. This discovery highlights the rich, yet under documented, marine biodiversity of Taiwan and underscores the value of combining traditional morphological examination with molecular phylogenetics for accurate species delimitation in cryptic nudibranch lineages.

## Introduction

The sea-slug genus *Thecacera* Fleming, 1828 belongs to the family Polyceridae Alder & Hancock, 1845, in the superfamily Polyceroidea, and represents one of the most distinctive groups of dorid Nudibranchia (Fleming 1828). The genus is characterised by having a limaciform body plan, truncated head with lateral extensions of the oral veil, and paired rhinophores situated within incomplete rhinophoral sheaths (Willan 1989). Since its establishment by Fleming in 1828 with *Thecacera pennigera* (Montagu, 1813) as the type species, the genus has grown to include six valid species distributed across temperate and tropical marine environments worldwide (Montagu 1813; Fleming 1828). Current valid species include *Thecacera boyla* Willan, 1989 from Australia, *Thecacera darwini* Pruvot-Fol, 1950 from Chile, *Thecacera pacifica* (Bergh, 1844) from the Pacific (Bergh 1884), *Thecacera pennigera* with a cosmopolitan distribution (Montagu 1813), *Thecacera picta* Baba, 1972 from Japan (Baba 1972), and *Thecacera vittata* Yonow, 1994 from the Indian Ocean (Yonow 1994).

Traditional morphology-based taxonomy has proven challenging for this group due to the presence of cryptic species complexes and considerable intraspecific variations in external characters (Carmona et al. 2014). Recent molecular phylogenetic studies have revealed hidden diversity within several genera of Nudibranchia, emphasising the importance of integrative taxonomic approaches that combine morphological and molecular data for accurate species delimitation (Dayrat 2005).

The western Pacific region, including the waters around Taiwan, represents a biodiversity hotspot for marine gastropods, yet the nudibranch fauna of this region remains poorly documented compared to other marine biogeographic provinces (Hoeksema 2007). Taiwan is located at the northernmost corner of the Indo-Pacific coral triangle that creates diverse marine habitats that support rich invertebrate communities (Jan et al. 2002). Despite this high diversity potential, systematic surveys of Nudibranchia in Taiwanese waters have been limited, and many taxa remain undescribed. During recent collecting expeditions in north-eastern Taiwan, specimens of an unknown *Thecacera* species were encountered on bryozoans. Preliminary morphological examination revealed distinctive characters that distinguished these specimens from all previously described *Thecacera* species. Subsequent molecular analysis using standard DNA barcoding markers confirmed the taxonomic distinctiveness of this population, providing strong evidence for its recognition as a new species.

This study describes *Thecacera sesama* sp. nov. based on a comprehensive morphological examination and molecular phylogenetic analyses. This work contributes to our understanding of nudibranch diversity in the western Pacific and provides important baseline data for future biogeographic and evolutionary studies of the genus *Thecacera*. The discovery also highlights the continued importance of taxonomic research in documenting marine biodiversity, particularly in understudied regions where many species await scientific description.

## Materials and methods

### Specimen collection and preservation

Six specimens of Nudibranchia were collected during multiple diving expeditions conducted between May 2021 and June 2025 at a depth of 18–30 m in Mother Rock Bay (25°12.09'N, 121°90.02'E, WGS 84, location marker 82.5 km on the Taiwan Provincial Highway No. 2) in northeastern Taiwan (Table 1). Diving is prevented in the north-eastern coast of Taiwan from October to April due to the low temperatures and strong waves, while the number of diving trips between May and September in this area is affected by typhoons. All specimens were collected using scuba diving and immediately photographed in situ to document living colouration and behaviour. According to the administering authority, the New Taipei City Marine and Fisheries Management Office, this site is not a protected area and therefore no permission is required for collection.

**Table 1.** Details for *Thecacera sesama* sp. nov. and its associated bryozoan specimen collected at the type locality and examined in this study.

Collection date	Depth (m)	Species	Type	GenBank accession number		Voucher
				COI	16S	
04 May 2021	21	*T. sesama*	Paratype	PX408749	PX410093	ASIZM 0001721
02 Aug 2021	25	*T. sesama*	Paratype	PX408750	PX410094	ASIZM 0001722
09 Sep 2021	19	*T. sesama*	Paratype	PX408751	PX410095	ASIZM 0001723
01 Jun 2025	28	*T. sesama*	Paratype	—	—	ASIZM 0001724
14 Jun 2025	23	*T. sesama*	Holotype	—	—	ASIZM 0001725
14 Jun 2025	30	*T. sesama*	Paratype	—	—	ASIZM 0001726
01 Aug 2025	18	Bryozoan	—	PZ246328	—	ASIZM 01000048

Following their collection, specimens were relaxed in a solution of magnesium chloride (MgCl_2_) in seawater to prevent muscular contraction during fixation. Tissue samples for molecular analysis were excised from the posterior portion of the foot and preserved in 95% ethanol solution. According to the specimen collection requirements of the Academia Sinica, the remaining specimens were fixed in 95% ethanol solution for long term preservation. All preserved specimens were deposited in the collection of the Biodiversity Research Centre, Academia Sinica (**ASIZM**).

### Morphological examination

External morphology was documented through detailed photography of living specimens in natural habitat conditions and under laboratory microscopic conditions. Measurements of body lengths, widths, heights, and spot lengths were taken from specimen photographs using ImageJ software. Morphological characters examined included body size, colouration, spots, rhinophores, rhinophoral sheaths, gills, post-branchial appendages, and tail (Smith 2024). Comparative morphological analysis was conducted using published descriptions and illustrations of all valid *Thecacera* species.

### DNA extraction, PCR, and sequencing

DNA samples from three specimens were extracted using a DNA extraction kit (Geneaid, cat. no. GS100). The mitochondrial cytochrome c oxidase subunit I (COI) and the 16S rRNA genes, which are widely used for resolving phylogenetic relationships and species delimitation in nudibranch, were chosen as the barcoding segments in this study. These genes were amplified by pairs of primers previously described (Palumbi 1991; Folmer et al. 1994). To amplify the barcoding segments, a polymerase chain reaction (PCR) mixture comprising 5–50 ng of template DNA, 12.5 μL of 2 × Taq PCR MasterMix (Genomix, GN-PCR201-01), and 12.5 μmol of each forward and reverse primer was prepared with distilled water to a final volume of 25 μL. The thermal cycling protocol included one cycle at 95 °C for 4 min, followed by 35 cycles of denaturation at 95 °C for 30 s, annealing at 45–50 °C for 30 s, extension at 72 °C for 30 s, and a final extension step at 72 °C for 7 min. Sequencing was carried out by Genomics Biotech Inc., Taipei, Taiwan, using the forward primer employed in PCR. The ends of amplified sequences were trimmed using CodonCode Aligner v. 10.0.1. The resulting barcode sequences from this study have been deposited to GenBank.

### Phylogenetic analysis

Returned DNA sequences were edited and assembled using BioEdit v. 7.2. Species identities for COI and 16S rDNA sequences were verified via BLAST searches against GenBank. Multiple sequence alignments were generated with MUSCLE implemented in MEGA v. 12 (Kumar et al. 2024). The COI dataset comprised 44 sequences (680 bp), and the 16S rDNA dataset included 29 sequences (527 bp). *Bathydoris clavigera* was designated as the primary outgroup for both COI and 16S phylogenetic reconstructions, based on its phylogenetically distant position relative to the studied Polyceridae taxa. *Vayssierea* sp. was retained in the dataset as an additional reference lineage, providing broader context for basal divergence patterns without serving as the operational outgroup for tree rooting. Phylogenetic relationships were inferred using both maximum likelihood (ML) and Bayesian inference (BI). Model selection using MEGA v. 12 identified GTR+I+G as the best-fitting nucleotide substitution model for both markers. ML analyses were executed in MEGA v. 12 with 1,000 bootstrap replicates under the GTR model with gamma-distributed rates and invariant sites, employing a 5-category discrete gamma distribution, subtree-pruning–regrafting (SPR) heuristic search, and initial trees obtained via maximum parsimony (Pola et al. 2023). Bayesian analyses followed standard MCMC parameters to ensure convergence and stable posterior probability estimation.

Bayesian analysis was performed in BEAST v. 1.10.4 with prior settings in BEAUti v. 1.10.4 (Suchard et al. 2018): general time reversible (GTR) as the substitution model, Sites as the site heterogeneity model, estimated base frequency, number of gamma categories set at 4, a strict clock, Coalescent: Constant Size as the speciation model, running 15 million generations for COI and 10 million generations for mitochondrial 16S rDNA with parameters and trees sampled every 1,000 generations. The first 25% of the trees were discarded as the burn in based on the effective sample size (ESS) of the parameter statistics in Tracer v. 1.7.2 (Rambaut et al. 2018), and posterior probabilities were calculated from the remaining samples. A summary tree was produced in TreeAnnotator v. 1.10.4 and viewed and edited in FigTree v. 1.4.4 (https://github.com/rambaut/figtree/releases; Suchard et al. 2018).

### Ecological observations

Behavioural and feeding data were recorded in the field. Prey species identification was based on examination of bryozoan colonies found in association with *Thecacera sesama* sp. nov.

## Results

### Systematic account

**Phylum Mollusca Cuvier, 1797**

**Class Gastropoda Cuvier, 1795**

**Subclass Heterobranchia J.E. Gray, 1840**

**Order Nudibranchia Cuvier, 1817**

**Superfamily Polyceroidea Alder & Hancock, 1845**

**Family Polyceridae Alder & Hancock, 1845**

**Subfamily Polycerinae Alder & Hancock, 1845**

**Genus *Thecacera* Fleming, 1828**

#### Thecacera sesama

Taxon classificationAnimaliaNudibranchiaPolyceridae

Chan & Lee sp. nov.

0565D6F2-52F5-5D5B-9457-D80709BD65C6

https://zoobank.org/C7586E98-D5AF-44A0-BA71-F3045D3D6E65

Figs 1, 2

##### Type material.

***Holotype***. • ASIZM0001725, 2.02 mm preserved length, collected 14 June 2025, 82.5 km, off Northern Coastal Highway, Ruifang District, New Taipei City, Taiwan (25°12.09'N, 121°90.02'E, WGS 84), 23 m depth, on bryozoan on the reef, collected by Ho-Yeung Chan. ***Paratypes***. • ASIZM0001721 to ASIZM0001724 and ASIZM0001726, 0.9–2.95 mm length, collected 04 May 2021, 02 Aug 2021, 09 Sep 2021, 01 Jun 2025, and 14 Jun 2025, 82.5, 19–30 m depth, bryozoan on the reef, collected by Ho-Yeung Chan (Table 1).

##### Diagnosis.

*Thecacera sesama* sp. nov. is distinguished by a unique combination of the following external morphological characters: (1) maximum preserved length of specimens is 2.83 mm; (2) body colour is translucent whitish, allowing some internal organs to be faintly visible; (3) entire body, as well as the rhinophores, rhinophoral sheaths, gills, post-branchial appendages, propodial tentacles, and tail are covered with numerous, discrete, small, circular, black spots and large yellow spots, as well as many white, snowflake-shaped pigment patches scattered on the body; (4) rhinophores and rhinophoral sheaths are translucent whitish, with small black spots and large yellow spots; (5) rhinophoral lamellae number 9–12; (6) gills number 5 and are translucent whitish, and the branchial plumes are pinnate; (7) post-branchial appendages are translucent whitish; (8) the head is translucent whitish, with short, blunt propodial tentacles at the corners.

##### Description

**Figure 1.**
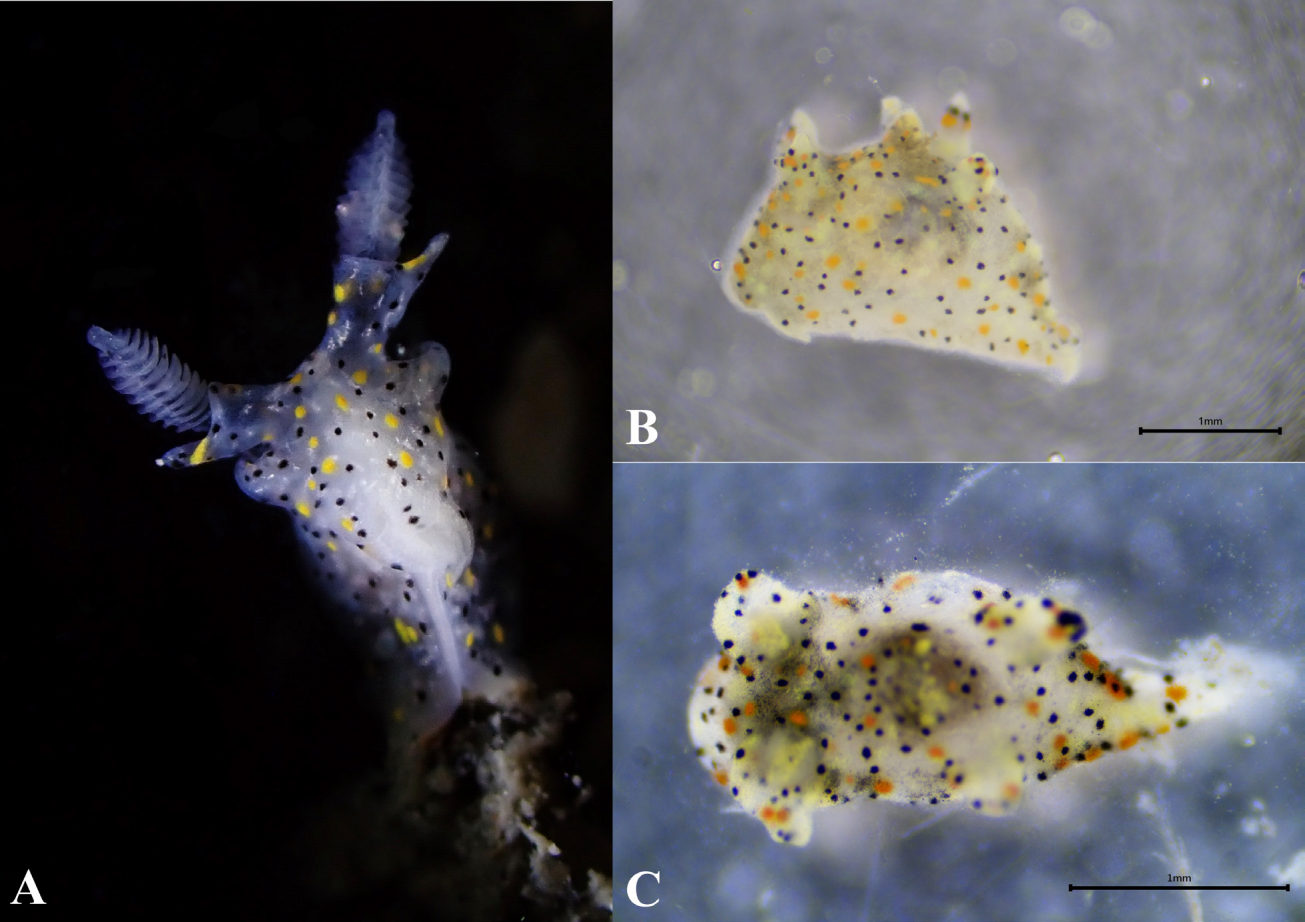
Living specimens of *Thecacera sesama* sp. nov. **A**. Ecological photos; **B**. ASIZM0001722; **C**. ASIZM0001721. Scale bars: 1 mm.

**Figure 2.**
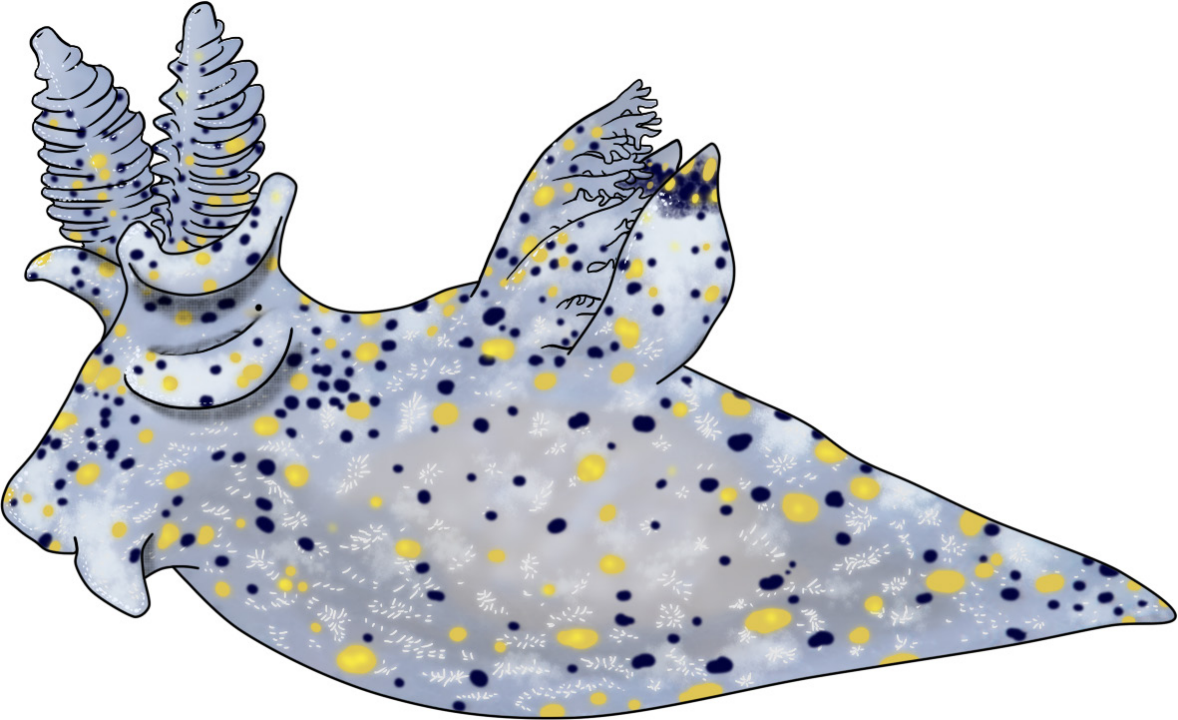
Details of appearance and morphological features, hand-drawn on a tablet PC by C-LL. *Thecacera sesama* sp. nov. Abbreviations: R rhinophores; RS rhinophoral sheaths; EA extra-rhinophoral appendages; PT propodial tentacles; G gills; PBA post-branchial appendages; T tail. Scale bar: 1 mm

**(Figs 1, 2)**. Body and size. This is a small species of *Thecacera* that has the body covered with a snowflake-like pattern of pigments. Body length 0.9–2.95 mm (*n* = 6, mean = 2.34 mm), width 0.63–1.11 mm (*n* = 3, mean = 0.94 mm) and height between 0.47–1.89 mm (*n* = 5, mean = 1.27 mm) (Table 2).

**Table 2.** Morphological data for the measured specimens in millimetres.

Voucher	Body length	Body width	Body height	Black spot	Yellow spot
ASIZM0001721	2.83	1.08	No record	0.05	0.07
ASIZM0001722	2.60	1.11	1.80	0.05	0.11
ASIZM0001723	2.77	No record	1.89	0.10	0.15
ASIZM0001724	2.02	No record	1.24	0.07	0.09
ASIZM0001725*	0.90	No record	0.47	0.03	0.07
ASIZM0001726	2.95	0.63	0.98	0.05	0.13
Mean	2.34	0.94	1.27	0.06	0.10

*Holotype. All other specimens are paratypes.

***Colouration***. The ground colour of all entire specimens is uniformly translucent white. This translucency allows the pale yellowish-white visceral mass to be visible through the dorsal integument.

***Spots***. The most striking characteristic of this species is the dense coverage of discrete, circular, small, black spots between 0.03 and 0.10 mm (*n* = 6 and mean = 0.06 mm) in diameter and larger yellow spots between 0.07 and 0.15 mm (*n* = 6 and mean = 0.10 mm) in diameter. These two types of spots are evenly distributed over the entire body. The spots do not coalesce (Table 2).

***Head and propodial tentacles***. The head is not clearly demarcated from the main body. At each anterolateral corner of the veil is a short, blunt, digitiform propodial tentacle.

***Rhinophores***. They emerge from cylindrical sheaths with smooth, slightly flared rims. The sheaths share the same translucent white ground colour and dense, small black and large yellow spotting as the body. The club bears 9–12 lamellae (*n* = 6 specimens and mean lamellae = 10). The club is translucent white.

***Extra-rhinophoral appendages***. The inner surfaces of the extra-rhinophoral appendages processes are fimbriate.

**Gills**. The branchial apparatus is situated dorsally in the posterior half of the body surrounding the anterior and lateral sides of the anal papilla. The five gills are entirely translucent white, although some specimens present a few small black spots and larger yellow spots on the outer base of the gill rachis.

***Post-branchial appendages***. A single, large, unbranched digitiform appendage is located on each side of the dorsum, positioned posterolaterally to the gill arch. These appendages are prominent, often held erect or angled dorsolaterally. They are structurally simple, sharing the body’s ground colour and dense pattern of small black spots and big yellow spots. The distal tip of some specimens is marked with conspicuous, large black spots.

***Foot***. The foot is translucent white. It is approximately the same width as the body, with a posterior that tapers to a blunt point, extending slightly beyond the dorsum.

##### Etymology.

The specific epithet *sesama* is derived from the Latin word for sesame seed, referring to the characteristic small, rounded, seed-like spots that cover the dorsal surface of this species, resembling scattered sesame seeds on the animal’s body.

##### Ecology and behavior.

**Figure 3.**
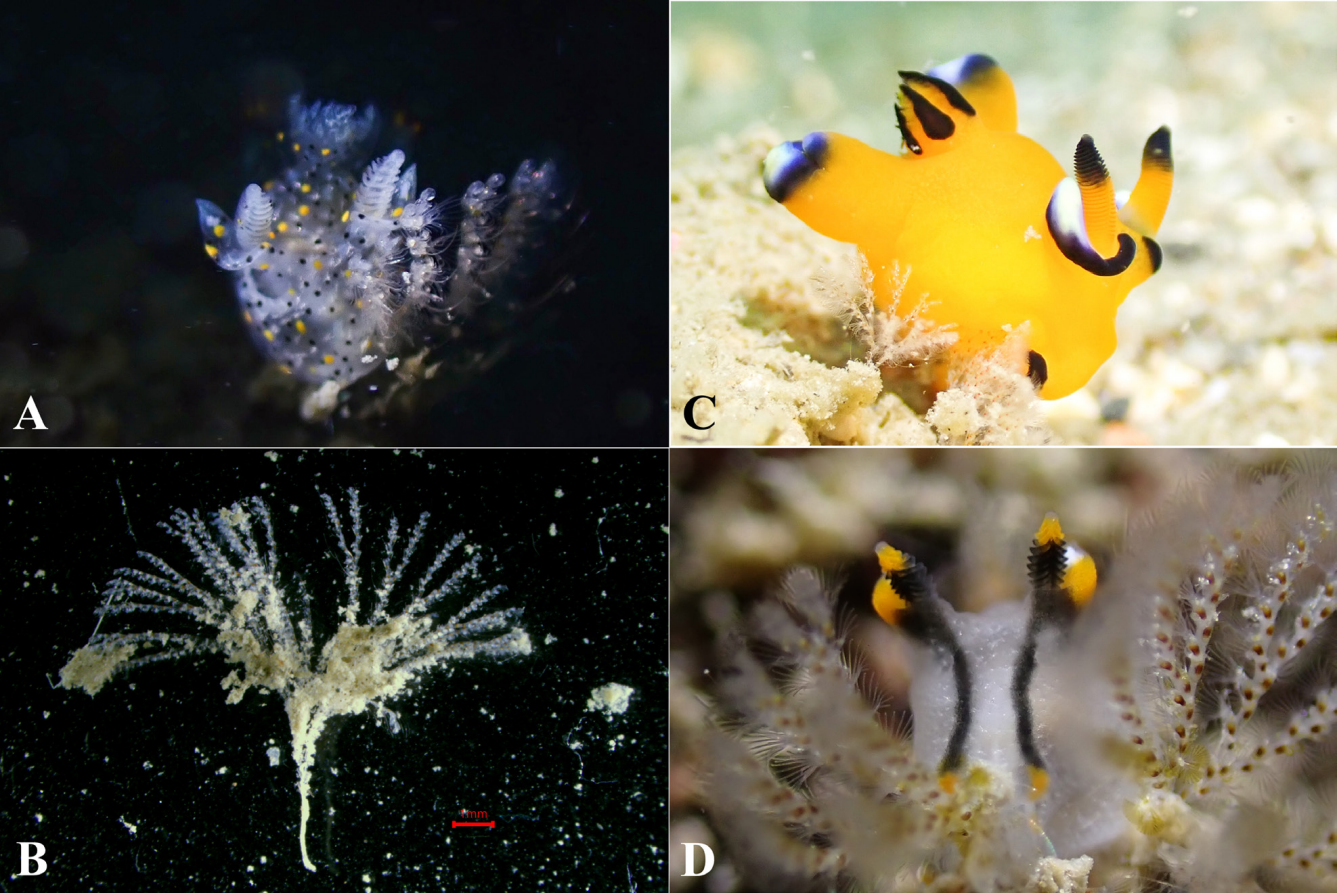
Living specimens of bryozoan with *Thecacera* species. **A**. *T. sesama* sp. nov. feeding on a bryozoan; **B**. Bryozoan, ASIZ0100005; **C**. *T. pacifica* feeding on a bryozoan; **D**. *T. picta* feeding on another bryozoan. Scale bar: 1 mm (**B**).

*Thecacera sesama* sp. nov. is a specialised predator of bryozoans, specifically observed feeding on one species of bryozoan (Fig. 3). Based on eight years of diving experience at the collection site from 2017–2025, *T. sesama*, *T. pacifica*, *T. picta*, and five undescribed *Thecacera* species were observed to feed on only two species of bryozoan, and these two bryozoans have significantly different appearances. The COI gene of the prey of *T. sesama* was sequenced and had low sequence similarities with the top sequence matches in the NCBI database, including *Watersipora edmondsoni* Soule & Soule, 1968 (82.31%) and *Bugula neritina* Linnaeus, 1758 (81.98%) (Table 3). After a morphological comparison between the prey of *T. sesama* and *W. edmondsoni*/*B. neritina*, significant morphological differences were observed between these species, and so the prey is referred as a bryozoan in this paper. The same bryozoan was only seen in the illustration of *Thecacera* sp. 3 (Nakano 2018), but it was not identified. The species exhibits typical *Thecacera* feeding behaviour, rasping the bryozoan colonies. Specimens are typically found in close association with their bryozoan prey, often positioned on or immediately adjacent to active colonies (Fig. 3, Table 4).

**Table 3.** Comparison of feeding habits among valid species of the genus *Thecacera*.

Species	Feeding habits	Reference
*T. sesama* sp. nov.	Bryozoan	This study
*T. darwini*	*Beania magellanica*	Marcus 1959: 59; Fischer et al. 2005
*T. pacifica*	Bryozoan	This study
*T. pennigera*	*Bugula flabellata* and *Bugula neritina*	Thompson and Brown 1984: 71; Mohamed Hatha 2017
*T. picta*	Bryozoan	This study

**Table 4.** Comparative morphology between similar species of *Thecacera*. ^a^ (Fischer et al. 2005); ^b^ (Nimbs et al. 2016); ^c^ (Baba 1960); ^d^ (Edmunds 2010); ^e^ (Jung et al. 2013); ^f^ (Yonow 1994); ^g^ (Kumar et al. 2019); ^h^ (Gosliner et al. 2008.).

	*Thecacera sesama* sp. nov.	*Thecacera darwini*	*Thecacera pacifica*	*Thecacera pennigera*	*Thecacera picta* (striped)	*Thecacera picta* (spotted)	*Thecacera vittata*
Maximum body length (mm)	2.83	45 ^a^	30 ^b^	25 ^c^	20	20	8.5 ^f^
Body colour	Translucent white	Translucent white ^a^	Bright orange ^g^	Translucent white ^c^	Translucent cream to grey	Translucent cream to grey	Translucent cream with black, orange, and white markings ^f^
Spots	Large yellow interspersed with smaller black circular spots	Black circular spots	None	Large orange interspersed with smaller black circular spots	Small amount, streaks, and continuous lines	Numerous and discrete circular spots	Black spots form distinct lines ^f^
Rhinophores	Translucent white with large yellow and small black spots	Light yellow	Orange with black on the top ^h^	Whitish ^c^	Black with orange at the tips	Black with orange at the tips	Black with orange at the tips
Rhinophoral sheaths	Translucent white with large yellow and small black spots	Dark yellow	Black, blue, and white tips	Whitish with big orange and small black spots	Black and orange tips	Black and orange tips	Black and orange tips
Lamellae	9–12	15–18 ^a^	No record	4–10 ^d^	No record	No record	15–19 ^f^
Gills colour	Translucent white with large yellow and small black spots	Whitish rachis and apices yellow ^a^	Orange with black on the top	Whitish ^c^	Translucent often with black line	Translucent often with black line	Translucent with black line
Post-branchial appendages	Translucent white with large yellow and small black spots	Yellow with white on the top	Black, blue, and white tips ^h^	Numerous big orange and small black spots scattered ^e^	Black, orange, and white tips	Black, orange, and white tips	Black, orange, and white tips
Tail colour	Translucent white with large yellow and small black spots	White and yellow tips	Black, blue, and white tips ^h^	Numerous big orange and small black spots scattered ^e^	Black and orange tips	Black and orange tips	Bright orange
Distribution	Indo-Pacific	Chile	Widespread in Pacific	Cosmopolitan	Widespread in Pacific	Widespread in Pacific	Maldives

##### Molecular characteristics.

DNA sequences were successfully obtained for both COI and 16S rRNA genes from three paratype specimens. The extremely small size of the specimens (0.9–2.95 mm) means that the whole body was used for DNA extraction. Therefore, only three paratype specimens were examined to ensure the preservation of the remaining specimens for long-term storage in museums. The COI sequences (658 bp) showed 88.27% similarity to *Thecacera picta* (GenBank accession no. KP871652), the closest match in GenBank databases. Intraspecific variations in COI sequences among the three specimens are minimal (0.2–0.4%), while their interspecific divergence with *T. picta* is substantial (14.17%). The 16S rDNA sequences (387 bp) of the new species had a 98.44% similarity to *T. picta* (GenBank accession KP871701), and have intraspecific variations of 0.3–0.6%, and interspecific divergence of 6.72% with *T. picta*.

**Figure 4.**
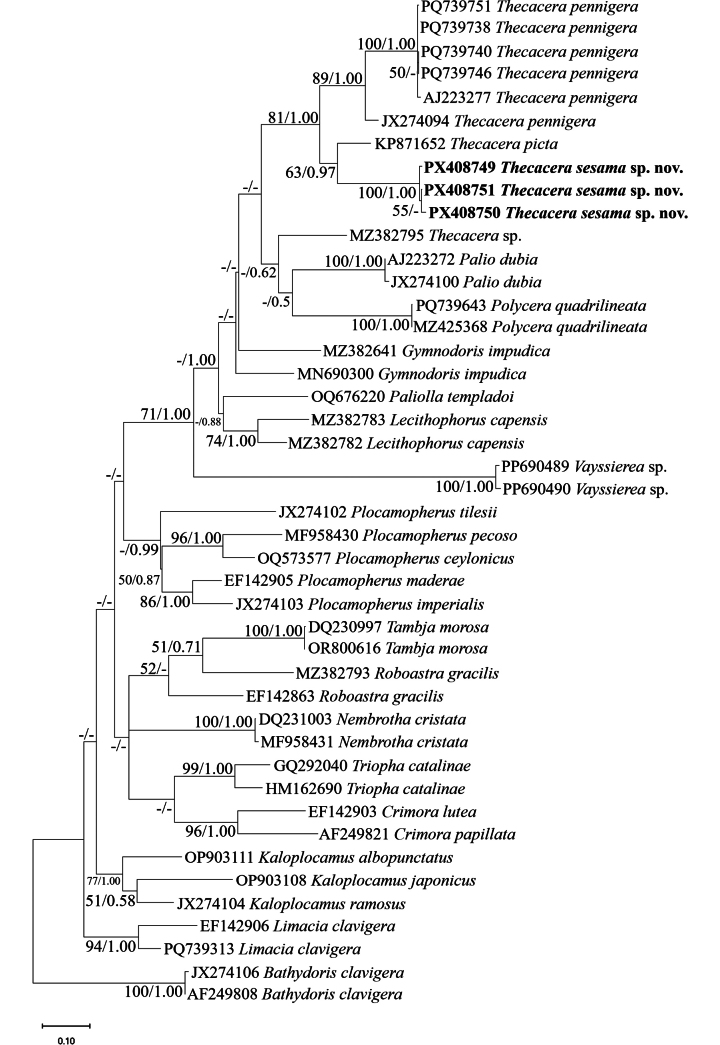
Maximum-likelihood (ML) phylogenetic tree inferred from mitochondrial cytochrome c oxidase subunit I (COI) gene sequences showing the relationship of *Thecacera sesama* sp. nov. and related species within the family Polyceridae. Bootstrap support values (left) and Bayesian posterior probabilities (right) are indicated at each node. The newly species is highlighted in bold. The scale bar represents 0.10 substitutions per site. *Bathydoris clavigera* was used as the outgroup.

**Figure 5.**
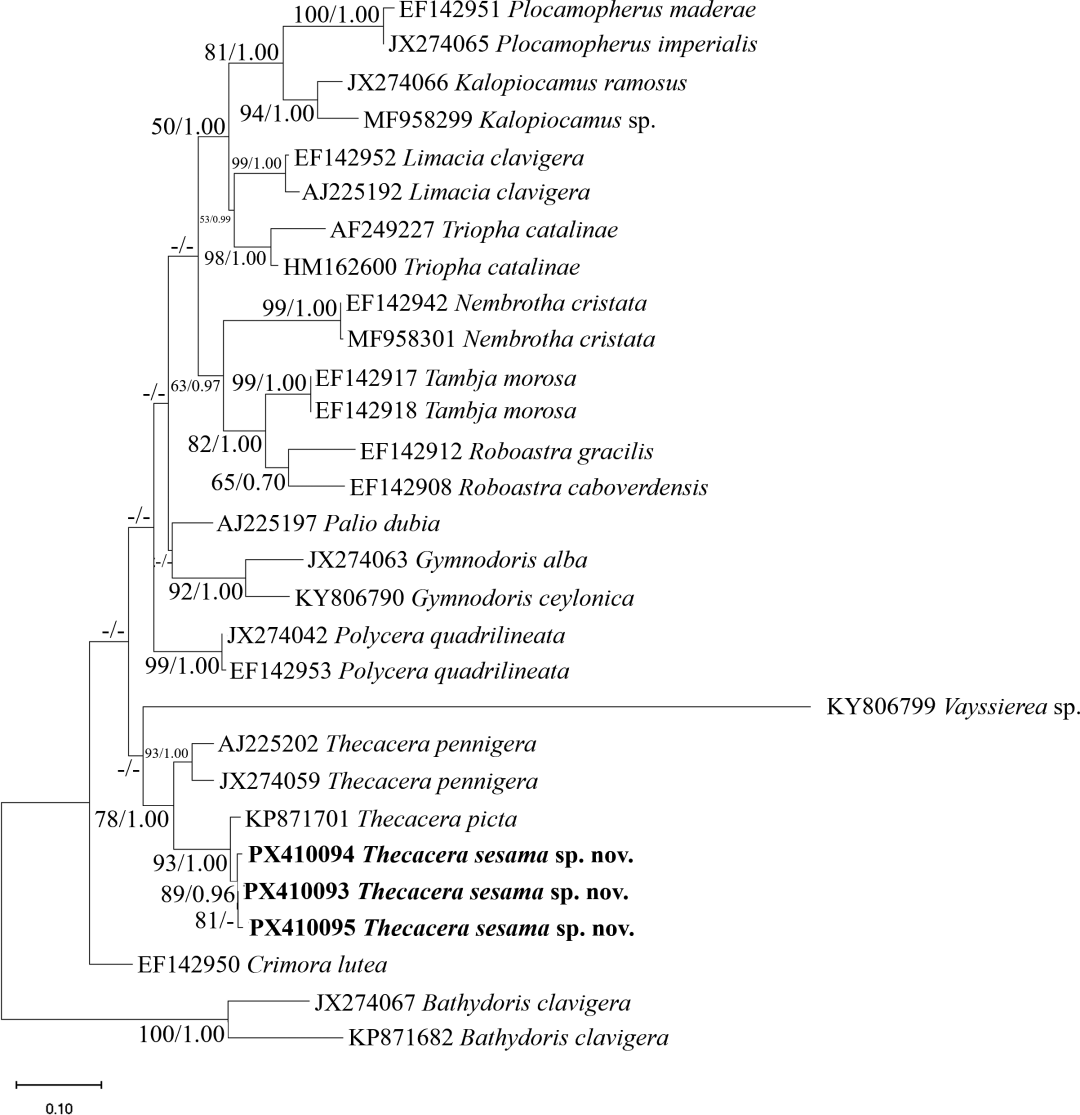
Maximum-likelihood (ML) phylogenetic tree based on mitochondrial 16S rRNA gene sequences showing the relationship of *Thecacera sesama* sp. nov. and related taxa within the family Polyceridae. Bootstrap support values (left) and Bayesian posterior probabilities (right) are indicated at each node. The new species is highlighted in bold. The scale bar represents 0.10 substitutions per site. *Bathydoris clavigera* was used as the outgroup.

**Molecular phylogenetic analysis**. The phylogenetic analysis based on COI sequences confirmed the taxonomic placement of *Thecacera sesama* sp. nov. within the genus *Thecacera*. The maximum-likelihood tree showed *T. sesama* forming a well-supported monophyletic clade (100/1.00, maximum-likelihood bootstrap (BS)/Bayesian posterior probability (PP) that is sister to *T. picta* with moderate support (63/0.97) (Fig. 4). Analysis of 16S rRNA sequences provides additional support for the species-level distinction of *T. sesama*. The 16S phylogeny shows a similar topology to the COI tree, with *T. sesama* specimens forming a strongly supported clade within *Thecacera* (78/1.00) (Fig. 5).

## Discussion

The description of *Thecacera sesama* sp. nov. represents an important addition to our understanding of nudibranch diversity in the western Pacific region, particularly of Taiwan. This new species brings the total number of valid *Thecacera* species to seven, and this is the first formal description of a member of the genus in nearly three decades, despite extensive research on the sea-slug biodiversity in the world’s oceans during the past three decades.

The initial nucleotide BLAST analysis of the COI sequences against the GenBank database confirmed that *T. sesama* represents a previously unknown lineage within *Thecacera*. The closest matches were *T. picta* sequences from Japanese waters, with maximum similarity values of 85.83%. No sequences with higher similarity were found in public databases, supporting the novelty of this taxon. The genetic distance between *T. sesama* and *T. picta* (14.17% for COI) is within the typical threshold for species level divergence in Nudibranchia (10–15%), suggesting an interspecific rather than intraspecific variation (Hebert et al. 2003). The 16S rDNA genetic distance between *T. sesama* and *T. picta* (6.72%) is also consistent with interspecific divergence levels observed in other genera of Nudibranchia (Puillandre et al. 2012). The substantial genetic divergence observed in these two genes exceeds typical intraspecific variation levels and falls within the range expected for closely related but distinct species in nudibranch taxa (Valdés 2003). Intraspecific genetic variation within *T. sesama* is minimal for both gene regions (COI: 0.2–0.4%; 16S rDNA: 0.3–0.6%), indicating genetic cohesion among specimens from the type locality despite collections during a 4-year period. This low intraspecific variation contrasts sharply with the substantial interspecific divergence from other *Thecacera* species, providing strong molecular evidence for the taxonomic validity of *T. sesama* as a distinct species. Phylogenetically, *T. sesama* is most closely related to *T. picta*, in a clade that also comprised the type species *T. pennigera*. Morphologically, *T. picta* does not possess the yellow spots on its body. *T. pennigera* is similar to *T. sesama* in their body colouration and spots, but the former species reach up to 30 mm (Pola et al. 2023). Other undescribed species of *Thecacera* also differ morphologically from *T. sesama*.

The distinctive morphological characteristics of *T. sesama* clearly distinguish it from all known *Thecacera* species. The close phylogenetic relationship between *T. sesama* and *T. picta* is consistent with their similar body shapes, although *T. picta* has individual differences in the black spots. The appearance and morphological characteristics of juvenile *T. picta* are similar to those of *T. sesama*. However, the clear differences in spots and colour, combined with the substantial genetic divergence, support their recognition as distinct species rather than geographic variants of a single widespread taxon (Table 4). The distinction between *T. sesama* and *T. picta* relies on specific spot colours, tip colour of the rhinophores, and post-branchial appendage tip colour. *Thecacera picta*’s rhinophores, rhinophoral sheaths, and post-branchial appendages are primarily black with orange edging. In contrast, *T. sesama*’s rhinophores, rhinophoral sheaths, and post-branchial appendages are transparent white, with small black and large yellow spots and no orange edging. *Thecacera sesama* and *T. pennigera* share similar morphological characteristics, but the new species is extremely small (<3 mm long), while *T. pennigera* is much bigger (reaching up to 25 mm long).

**Figure 6.**
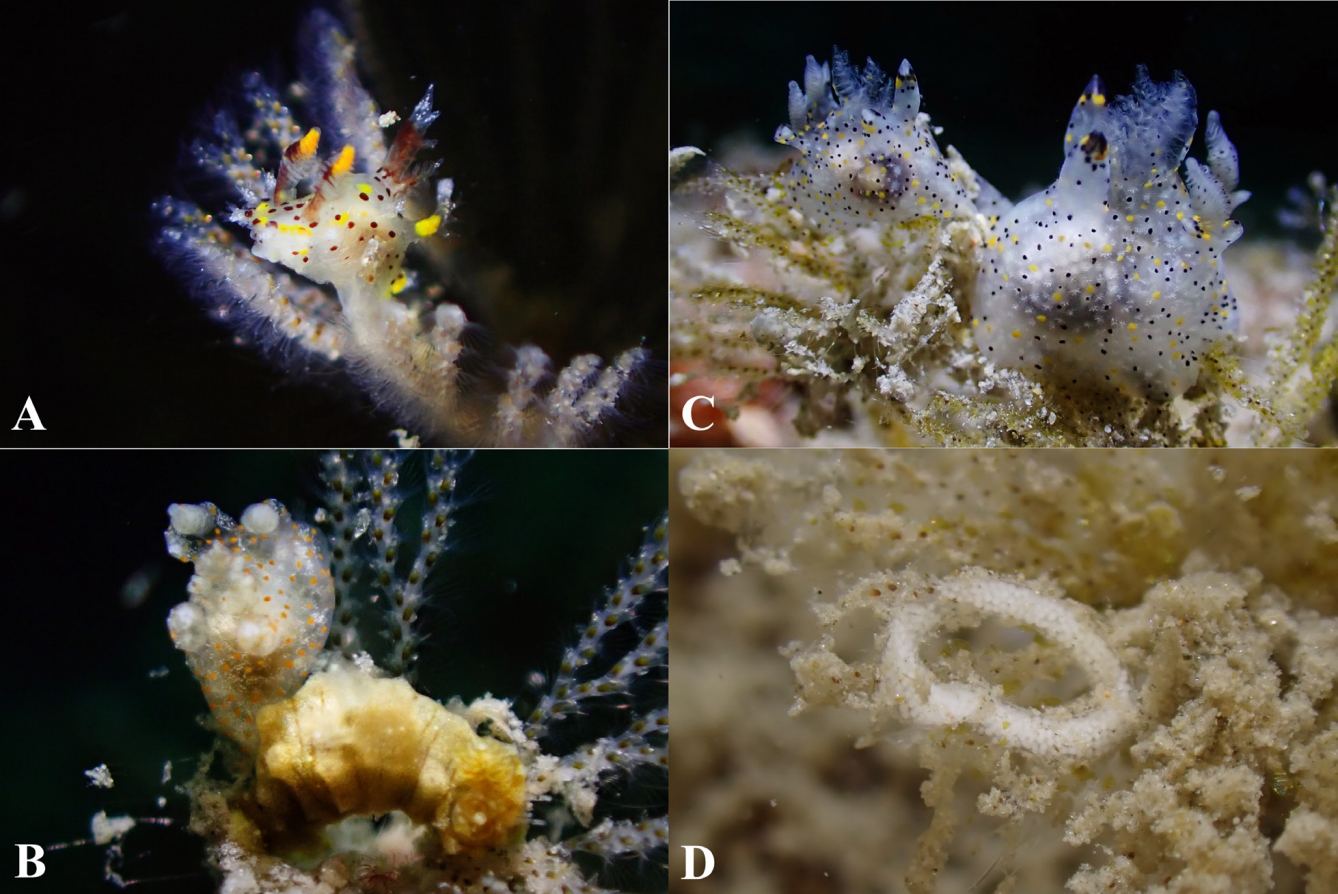
Polyceridae associated with bryozoans. **A**. *Plocamopherus tilesii* feeding on a bryozoan; **B**. Associations between species sharing a food source: an undescribed *Thecacera* sp. (left) and *Polycera risbeci* (right) co-occurred and fed on the same bryozoan; **C**. Two individuals of *Thecacera sesama* sp. nov. feeding on a bryozoan; **D**. Egg ribbon of a polycerid species.

The ecological specialisation of *T. sesama* on its bryozoan is consistent with the feeding ecology of other *Thecacera* species, which are known to be specialised predators of various bryozoan taxa (Todd 1981). The co-occurrence of *T. sesama* with its bryozoan prey at the type locality suggests a close ecological relationship that may influence the species’ distribution and abundance patterns. The discovery of *T. sesama* in Taiwan highlights the high species diversity within the family Polyceridae, particularly in the western Pacific biodiversity hotspot. In the same habitat where *T. sesama* occurs, other polycerids include species of the genera *Plocamopherus* and *Polycera*, as well as congeneric species like *T. pacifica* and *T. picta*, which were also observed feeding and laying eggs on the same bryozoan (Fig. 6). Given that these small nudibranchs are generally smaller than 30 mm in length and utilise identical resources, the resulting high species diversity within a limited ecological niche is remarkable. While *T. sesama* is currently only confirmed from north-eastern and Kenting National Park of Taiwan, its distribution potentially extends beyond Taiwan.

## Conclusions

The new nudibranch species *Thecacera sesama* sp. nov. is described from north-eastern Taiwan using an integrative taxonomic approach combining morphology and molecular data from the COI and 16S rRNA genes. Molecular phylogenetic analyses confirm the species is a distinct lineage, with the COI gene showing a significant interspecific divergence of 14.17% from its closest relative, *Thecacera picta*. Morphologically, *T. sesama* is uniquely distinguished by its translucent white body covered with numerous small, round, black pigment spots and fewer, larger yellow spots. The phylogenetic analysis places *T. sesama* as a sister species to *T. picta*.

## Supplementary Material

XML Treatment for Thecacera sesama

## Additional information

### Artificial Intelligence (AI) use

The authors accept full responsibility for the content of the manuscript, including the disclosure of any use of AI.

No AI tools were used in the preparation of this manuscript.

### Author ORCIDs

C.-H. Chang https://orcid.org/0000-0001-8566-3361

### Data availability

DNA sequences have been deposited in GenBank under accession numbers (PX408749, PX408750, PX408751, PX410093, PX410094, PX410095). Holotype and paratype are deposited in the collection of the Biodiversity Research Centre, Academia Sinica (ASIZM).
